# Low and unequal use of outpatient health services in public primary health care facilities in southern Ethiopia: a facility-based cross-sectional study

**DOI:** 10.1186/s12913-021-06846-x

**Published:** 2021-08-06

**Authors:** Hiwot Abera Areru, Mesay Hailu Dangisso, Bernt Lindtjørn

**Affiliations:** 1 School of Public Health, College of Medicine and Health Sciences, Hawassa University, P.O.BOX: 1560, Hawassa, Ethiopia; 2 Global Public Health and Primary Care, Centre for International Health, University of Bergen, Bergen, Norway

**Keywords:** Health service utilisation, Outpatient department visit, Service users’ involvement, Patient and public involvement, Health authorities, Diagnostic capacity, Sidama, Southern Ethiopia

## Abstract

**Background:**

Outpatient department visits per individual for each year are one of the core indicators of healthcare delivery to assess accessibility or quality of services. In addition, this study aimed to assess health service utilisation and disease patterns in southern Ethiopia, by including the health authorities’ suggestions to improve the services. No study has assessed this in Ethiopia previously.

**Methods:**

An institution-based cross-sectional design study was done in 65 primary health care units in Dale and Wonsho districts, in Sidama region, for all patients visiting health facilities from 1 July 2017 to 30 June 2018. We estimated the utilisation rate as visits per person per year, the odds ratio for health use and proportions of diseases’ diagnoses. The results of our study were presented to local health authorities, and their suggestions for improvements were incorporated into the analysis.

**Result:**

A total of 81,129 patients visited the health facilities. The annual outpatient health service utilisation was 0.18 (95% CI: 0.18–0.19) new visits per person per year. The health service utilisation rate per year for the rural population was lower than the urban utilisation by 91% (OR = 0.09; 95% CI: 0.08–0.09). Children in the age group of 5–14 years had lower odds of health service utilisation by 78% (OR = 0.22; 95% CI: 0.21–0.23), compared to children under 5 years of age. Females were four times (OR = 4.17; 95% CI: 4.09–4.25) more likely to utilise health services than males. Febrile illness constituted 17.9% (14,847 of 83,148) of the diagnoses in all age groups. Almost half of the febrile cases, 46.5% (3827 of 8233), were among children under 5 years of age. There were very few cases of non-communicable diseases diagnosed in the health facilities. The health authorities suggested improving diagnostic capacities at health centres, enhancing health professionals’ skill and attitudes, and improving affordability and physical accessibility of the services.

**Conclusion:**

The health service utilisation rate was low in Sidama. The use of health services was lower among rural residents, men, children and elderly, and health post users. Improving the quality, affordability and accessibility of the health services, by involving responsible stakeholders could increase service usage.

**Supplementary Information:**

The online version contains supplementary material available at 10.1186/s12913-021-06846-x.

## Background

According to the World Health Organization, “health services include all services dealing with the promotion, maintenance and restoration of health. They include both personal- and population-based health services” [1]. Half of the world’s people lack some or all essential health services [2]. Health service utilisation data can be obtained from national population surveys, health facility reports and routinely collected demographic and health surveys [3]. The health system indicators used to measure the health service utilisation in Ethiopia include, annual outpatient (OPD) attendance per individual, admission rates, bed occupancy rates and average length of stay [4].

Primary healthcare units constitute health centres and health posts, which are the foundation of the Ethiopian health system; primary hospitals are also included in the primary-level healthcare system [5]. Even though the Ethiopian health and health service system has improved in the last 20 years, most of the goals related to healthy lives and well-being have not yet been achieved [6–8]. Advances have been made in interventions like family planning, antenatal care, maternal healthcare, and hygiene and sanitation since the introduction of health extension workers who are deployed in health posts [9]. However, like other low- and middle-income countries, Ethiopia is still facing a triple burden of disease, with communicable diseases, non-communicable diseases and injuries being the main challenges [5]. The essential health service package of Ethiopia serves as a primary framework of achieving universal health coverage through quality primary health care services. The newly revised Ethiopian essential health service package included nine priority areas based on the burden of disease in the country. These includes reproductive, maternal, neonatal, child and adolescent health, major communicable diseases (HIV/AIDS, tuberculosis and malaria), non-communicable diseases, surgical and injury care, emergency and critical care, neglected tropical diseases, hygiene and environmental health services, health education and behaviour change communication services and multi-sectoral nutrition interventions [10].

Ethiopia aims at improving utilisation of healthcare services in an equitable and accessible manner [5]. Besides, universal health coverage aims to guarantee that all individuals get access to needed, important and adequate quality health services, while being protected from undue financial hardships [5, 11]. The Federal Ministry of Health has given priority to maternal and child health, and also to family planning, followed by malaria eradication [6, 7]. Although official documents suggest that the health service coverage of the country has reached 98%, the performance on major health indicators remains far below the targets set by the government [5, 8]. In addition, a recent study on universal health coverage showed a national coverage of 34%, with great regional variability [12]. Moreover, the national household health service utilisation survey showed that among people who reported being ill, only 53% visited health facilities. The common reasons for not seeking care were cost, proximity, self-medication and the thought of diseases as not series [13].

Outpatient service utilisation, as measured by the number of outpatient department (OPD) visits per person per year, is one of the recommended core indicators of health service delivery [14]. Low rates of outpatient visits are indicative of limited accessibility or low quality of services [15]. A study done in 130 countries in 2016 showed that the global outpatient age-standardised utilisation rate was 5.4 visits per individual per year [16]. In 2015, the average OPD visits rate in Ethiopia was 0.48 visit per person per year; however, the target was two visits per person per year by 2020 [5, 17]. The World Health Organization recommends around three to four OPD visits per person per year [18].

The rate and type of health service utilisation can be directly or indirectly affected by certain demographic attributes; these include age, sex, marital status, education, and income. The change that occurred in the health service utilisation pattern in the developed world, like the United States of America, is partly due to such changes in demographic characteristics of the population [19]. The same holds true in other Sub-Saharan Africa countries [20, 21].

Even though involving stakeholders, such as health service providers and authorities, has been a common practice in developed nations’ studies, it has only recently been given some attention in Africa [22, 23]. The combined effort of the service providers or health authorities, users and researchers can be useful in creating insight into the problems and provide a framework for remedial implementation, thereby improving the quality and impact of health research [23–25]. It is believed that health authorities will make decisions with policy implications after they critically evaluate the potential benefits for the improvement of primary health services [26]. Therefore, to ensure the implementation of research findings and bring change in legislation that can impact the quality of healthcare, it is mandatory to make such a process interactive and evidence-based, to build trust and understanding between the researchers and policymakers at local, regional and national levels [27, 28]. In this study, the stakeholders were given the result from their own facilities, and were asked to comment and suggest possible ways to improve the services at their institutions [29].

To our knowledge, in Ethiopia, there has been only one study 30 years ago that assessed the overall utilisation of health services as visits per individual per year. Besides this, there are no studies that presented the result of a study to the health authorities and included their opinions and recommendations as part of the investigation in Ethiopia. The objective of this study was to assess the health service utilisation and disease patterns in southern Ethiopia. In addition, we sought to get the local health authorities’ suggestions to improve the health services in the area.

## Methods

### Study design and period

The study employed an institution-based cross-sectional study design and included all patients visiting these public health centres and health posts from 1 July 2017 to 30 June 2018 (1 year).

### Setting

The Sidama region is one of the most densely populated areas in Ethiopia, with 533 persons/km^2^ [30, 31]. The region constitutes about 4.0% of the national population [32]. Agriculture is a prominent economic sector with ‘enset’ (*E. ventricosum*) and maize as the main foods, and coffee and fruits as the most important cash products. ‘Khat’, a form of chewable stimulant, is another cash crop widely produced [33].

We did the study in Dale and Wonsho districts (woredas). In 2017, the Dale district has a total population of 268,839 people and an estimated 53,768 households. It has 36 rural and two urban kebeles (the lowest administrative structures). Yirga Alem town is the main town in the district and has five urban kebeles. Wonsho district has 129,730 people living in 17 rural and one urban kebeles in 21,857 households [33, 34]. There are ten health centres and 33 health posts in Dale, and the Wonsho district has five health centres and 17 health posts [33, 34]. Even though there are two private clinics in rural Dale and one in Wonsho district, we believe most rural communities seek health services from governmental institutions [35]. Therefore, we studied all 65 governmental healthcare facilities available in Dale and Wonsho districts.

### Participants

All sixty-five primary health care units (15 health centres and 50 health posts) in Dale and Wonsho districts were included in our study. Moreover, the health officials, from local to regional health facilities and offices, participated in this study. Seven health professionals were selected purposively so that they could comment and suggest ways to improve the health services of the primary health care institutions. These health professionals were: one from each of the district health departments, one from rural health centres from each district, one from urban health centres from each district, and one health professional from the Sidama Regional Health Bureau.

### Study size

Assuming the national individual utilisation rate per year of OPD visit, as 48% prevalence, a power of 80, 95% confidence interval, a margin of error of 1.4% and the ratio of sample size in unexposed/exposed of 1, the required sample size was 4869 patients. The cases included in this study, however, were 81,129. Participants from health facilities and offices were selected purposively due to their position and involvement in decision-making processes.

### Variables and data collection procedure

The main outcome measures were visits to primary health care units per person per year and the proportions of diagnoses. The exposure variables were gender, age, residence (urban or rural), district, type of health facility, disease types and visit types (new or repeat).

Fifteen local data collectors with a diploma in nursing and two supervisors with BSc degrees were trained to collect the data from the institutional registries at each health centre and health post. Standard registers supplied by the Federal Ministry of Health to the health centres and health posts were reviewed. Each unit or department had registry with the registration, identification and service-related information. The lists of the registries and the major components reviewed are attached as Table 8, additional file 1.

The diagnoses made by the health workers in health centres were mainly clinically, using guidelines, such as Integrated Management of Childhood Illness (IMCI), and sometimes supported by laboratory services. The health extension workers made diagnoses based on the working manuals, such as Integrated Community Case Management (iCCM) of malaria, pneumonia and diarrhoea, and TB (tuberculosis) screening guidelines. Then, the diagnoses obtained from the registry of health facilities were categorised according to the national or international disease classification (ICD) during data cleaning [36, 37].

At three institutions, complete data couldn’t be found; in the delivery unit of Gidamo health centre, and the registration record for 5 months was not found. Similarly, in Bera health centre, data on delivering mothers for 5 months were missing. Besides, the information from the eye clinic at Bokaso health centre was not found because it was locked during the data collection period.

Since involvement of the health authorities was a secondary focus of this study, the Guidance for Reporting Involvement of Patients and the Public 2-short form were used as guides to collect, analyse and present the data from the health authorities [29]. After analysis, the results were presented to Dale and Wonsho district health office managers, and to the urban and rural health centre managers, and to the Sidama Regional State Health Bureau. They were informed about the results of our study and were asked to suggest areas of possible improvements. Two data collectors with BSc degree interviewed the respondents (one for each district). They were all encouraged to give their feedback based on the results and to suggest possible areas of improvements.

### Data analysis

The data collection instruments were pretested on health centres and health posts outside of both districts. The data collectors were trained for 2 days about the protocol and how to extract the data from the registries. Re-checking was done by the principal investigator on 5% of the cases in each health facility, to assure the quality of the data.

The data were double entered and validated in EpiData version 3.1 software (EpiData Association Odense, Denmark). The analysis was done by STATA software version 13 (Stata Corp., LLC. College Station, Texas, USA). Descriptive statistics were done for all cases and variables. Stratification was done based on background characteristics of the clients on service utilisation and disease diagnosis. We used the number of new visitors to calculate the odds ratio and 95% confidence interval and compare the rate of utilisation for each explanatory variable. The WHO core health indicators manual was used to calculate outpatient utilisation rate [14].

The denominators for the utilisation rate calculations were based on projections of the 2007 census done in Sidama [38]. Thus, we obtained the estimated population for the year 2017/18 for women, children under 5 years of age, infants (under 1 year of age), people older than 5 years of age, and sex ratio.

Based on these population estimates, we calculated the utilisation rates as new visits per person per year for each age group, sex, family planning, antenatal care, postnatal care, delivery, pneumonia, diarrhoea and malnutrition. Microsoft Excel and MedCalc Software version 19.2.6 (MedCalc Software Ltd., Ostend,Belgium) were used to calculate these rates. Tables and figures were used to display the findings.

Missing information was identified on three of the variables (visit type, sex and age). We coded the missing values as “not recorded”. Hence, the utilisation rate was calculated for new cases only.

Each of the health staff gave their feedback on the results. Their feedback was analysed manually. First, we read and re-read their responses to identify similar ideas and then we categorised them based on the emerging themes. Following this, we separately organised them as district and regional authorities’ responses under each themes. Finally, the quotes were organised under each theme in the summarised table.

#### Operational definitions

More definitions are attached in additional file 2.

Outpatient utilisation rate: The number of new outpatient visits to health facilities per year relative to the total population of the same geographical area.

We calculated the utilisation rates for each variable as: The number of disease events divided by the eligible population of the same geographic area. We estimated the proportion of eligible population by multiplying the total population of the districts by the percentage of a particular category obtained from census and surveys.

For annual immunisation utilisation rate the denominator was the cohort of children in a year. We divided the number of under-five year children in the study area by five to obtain a single year children since we collected the data for children immunised in 1 year.

New visitors were defined as those patients who attended the health facility for the first time. Repeat visitors were those who attended the health facility for multiple times for the same diagnoses or illness within the reporting period, one Ethiopian fiscal year (July to June) and registered once as new visitor [39].

For family planning service: “New acceptors” refers to the number of modern contraceptive method acceptors who receive family planning services from a recognised family planning providing facility for the first time irrespective of the method used. Each such acceptor was counted once. Each “repeat acceptor” is counted once, irrespective of number of times family planning services were received during that fiscal year [39].

For immunisation services, new visitors were those who received their first dose of vaccines and repeat visitors were those who received more than one dose in 1 year.

For antenatal care service those clients who present for the first follow-up visit in a year were considered as new visitors. Those clients who came for subsequent follow-ups were repeat visitors; however, they are counted only once.

## Results

### Background information

**Fig. 1:**
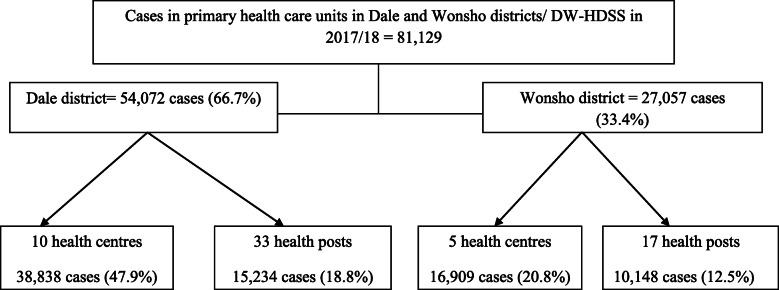
Flowchart of case distribution in Dale and Wonsho districts, 2017/18, Sidama, Ethiopia (*N* = 81,129)

More than two-thirds of the cases (69%) received service or treatment from health centres. Two-thirds (67%) of the cases were from Dale district (Fig. 1).

The total number of the clients who visited the health facilities was 81,129. Most of the clients were female (74%) and were from rural areas (82%).The mean age of the clients was 21.9 years (range: 0–120). About a third (37%) of the cases were in the age group of 25–34 years (Table 1).

**Table 1:** Background characteristics of clients visiting health facilities in Dale and Wonsho districts, 2017/18, Sidama, Ethiopia (*N* = 81,129)

Variables	Number of cases (***N*** = 81,129)	%
**Type of health facility**		
Health centres’ cases	55,747	68.7
Health posts’ cases	25,382	31.3
**Residence**		
Rural	66,341	81.8
Urban	14,788	18.2
**District**		
Wonsho	27,057	33.3
Dale	54,072	66.7
**Sex**		
Female	59,822	73.7
Male	20,232	25.0
Not recorded	1075	1.3
**Type of visit**		
New	73,513	90.6
Repeat	1048	1.3
Not recorded	6568	8.1
**Age group (in years)**		
Under 5	13,112	16.2
5–14	4788	5.9
15–24	17,729	21.9
25–34	30,172	37.2
35–44	5268	6.5
45–54	1754	2.2
55–64	826	1.0
65–74	335	0.4
75+	208	0.3
Age missing	6937	8.6

### Utilisation rates

The annual outpatient health service utilisation rate was 0.18 (95% CI: 0.18–0.19) for new visitors. The number of new visits per year for rural people was 0.16 (95% CI: 0.15–0.16) and 0.68 (95% CI: 0.67–0.69) for urban population. Therefore, the rural population visited health institutions 11 times less than urban people (OR = 0.09; 95% CI: 0.08–0.09). Children in the age group of 5–14 years had lower odds of health service utilisation by 78% (OR = 0.22; 95% CI: 0.21–0.23), when compared to children under 5 years. Similarly, the odds of health service utilisation was lower for people aged above 75 years by 69% (OR = 0.31; 95% CI: 0.27–0.36), compared to children under 5 years. Females were 4.17 (95% CI: 4.09–4.25) times more likely to utilise health services than males (Table 2).

**Table 2:** Utilisation rates by new clients’ characteristics in Dale and Wonsho district primary health care units (PHCUs), 2018, Sidama, Ethiopia (*N* = 73,513)

Variables	Utilised health service		Estimated total number of eligible population	% (out of 73,513 new cases)	Rates of utilisation (95%CI)	Odds ratio (95%CI)	***P***-value
	Yes	No					
**Health facilities**							
Health centres’ cases	50,275	348,294	398,569	68.7	0.13 (0.12–0.13)	2.33 (2.29–2.37)	*P* < 0.0001
Health posts’ cases	23,238	375,331	398,569	31.3	0.06 (0.05–0.06)	1	
**Residence**							
Rural	59,929	318,712	378,641	81.8	0.16 (0.15–0.16)	0.09 (0.08–0.09)	*P* < 0.0001
Urban	13,584	6345	19,929	18.2	0.68 (0.67–0.69)	1	
**Districts**							
Wonsho	25,066	104,664	129,730	34.1	0.19 (0.19–0.20)	1	*P* < 0.0001
Dale	48,447	220,392	268,839	65.9	0.18 (0.17–0.18)	0.92 (0.90–0.93)	
**Sex**							
Female	55,159	141,180	196,339	75.0	0.28 (0.27–0.28)	4.17 (4.09–4.25)	*P* < 0.0001
Male	17,334	184,896	202,230	23.6	0.09 (0.08–0.09)	1	
Not recorded	1020	–	–	1.4	–		
**Age group**							
Under 5	8936	53,293	62,229	12.2	0.14 (0.14–0.15)	1	
5–14	4508	124,039	128,547	6.1	0.04 (0.03–0.04)	0.22 (0.21–0.23)	*P* < 0.0001
15–24	17,125	59,678	76,803	23.3	0.22 (0.22–0.23)	1.71 (1.66–1.76)	*P* < 0.0001
25–34	28,816	25,178	53,994	39.2	0.53 (0.53–0.54)	6.83 (6.64–7.02)	*P* < 0.0001
35–44	4512	31,517	36,029	6.1	0.13 (0.12–0.13)	0.85 (0.82–0.89)	*P* < 0.0001
45–54	1536	19,036	20,572	2.1	0.07 (0.07–0.08)	0.48 (0.45–0.51)	*P* < 0.0001
55–64	762	9896	10,658	1.0	0.07 (0.07–0.08)	0.46 (0.43–0.50)	*P* < 0.0001
65–74	312	5624	5936	0.4	0.05 (0.05–0.06)	0.33 (0.29–0.37)	*P* < 0.0001
75+	189	3652	3801	0.3	0.05 (0.04–0.06)	0.31 (0.27–0.36)	*P* < 0.0001
Age missing	6817	–	–	9.3	–		
Total	73,513	331,873	398,569	100	0.18 (0.18–0.19)		

### Prevalence of maternal health services utilisation

The prevalence of family planning utilisation was 28.3% (22,384 family planning users divided by the number of fertile women in the districts which was 79,076 (398,569 *19.84%)). Most of the family planning service clients, 73.6% (16,481 of 22,384 clients), used short-acting contraceptives, which include Depo-Provera or medroxy progesterone acetate, contraceptive pills (combined or progesterone-only oral contraceptives), and condoms. Long-acting contraceptives, such as Intra-Uterine Contraceptive Device (IUCD) and implants, were used by 26.3% (5894 of 22,384 clients). Yet, only nine of 22,384 (0.04%) patients chose a permanent contraceptive method such as bilateral tubal ligation.

The utilisation of antenatal care service in this study was 45% (6206 antenatal care clients per an estimated 13,791 pregnant mothers in the districts (398,569 *3.46%)) and delivery service utilisation was 40.7% (5619 delivering mothers per an estimated 13,791 pregnant mothers in the districts). Postnatal care service utilisation in this study was 14.3% (1973 mothers and children who received postnatal care per an estimated 13,791 pregnant mothers in the districts).

### Treatment at departments

Of the departments that provided health services, 30% (24,388 of 81,129 cases) received service from the adult outpatient department. Family planning service was the next most utilised service, constituting 28% (22,423 of 81,129) of the cases. The stabilisation centre for malnutrition was the least common of the services, utilised by 33 of 81,129 (0.04%) of the visitors (Table 6 is attached as additional file 3).

### Caseloads/diagnosis

Table 3 shows that clients coming to get contraceptive services constitute 26.9% (22,384 of 83,149) of the caseload. Typhoid and typhus fever were the most common registered ailments, contributing to 5.9% (4931 of 83,149) of the diagnoses, with 92% (4210 of 4555 cases) above the age of fifteen; 57% of these were females (2604 of 4555 cases). From non-communicable diseases, hypertension was diagnosed in 0.4% (304 of 83,149) and 63% (190 of 304) of these were females.

**Table 3:** Recorded diagnoses by age group, sex, type of health facility and visit type of the cases in Dale and Wonsho districts primary health care units in 2018, Sidama, Ethiopia (*N* = 83,149)

Diagnosis	Number of diagnoses	%	Age group			Sex			Type of health facility		Type of visit			Total number of clients
			< 5 years	5–14 years	> 15 years	Male	Female	Missing	Health centre	Health post	New	Repeat	Missing	
Family planning	22,384	26.9	0	0	18,936	0	22,384	0	12,039	10,345	22,384	0	0	22,384
Immunisation	7549	9.1	4193	0	540	3188	3471	890	1197	6352	7549	0	0	7549
Antenatal care	6206	7.5	0	0	5878	0	6206	0	2915	3291	6206	0	0	6206
Delivery	5619	6.8	0	0	5600	0	5619	0	5619		5619	0	0	5619
Typhoid and Typhus fever	4931	5.9	6	337	4210	1948	2604	3	4931		4672	86	173	4555
Pneumonia	4072	4.9	2828	495	713	2071	1992	9	2735	1337	2695	240	1137	4072
Parasitic and helminthic infections	3660	4.4	287	840	2519	1790	1854	7	3620	40	3298	72	290	3651
Fever and febrile illness	3895	4.7	527	629	2709	1703	2181	11	3844	51	3363	97	435	3895
Upper and lower respiratory disorders	2930	3.5	877	487	1516	1446	1457	9	2645	285	2262	48	620	2912
Accident (burn, trauma, injury, road traffic accident)	2609	3.1	80	567	1952	1708	880	21	2608	1	2465	24	120	2609
Screening for cancer, human immunodeficiency virus and helminths	2567	3.1	78	2	2464	24	2540	3	2437	130	1148	0	1419	2567
Diarrhoea	2311	2.8	1371	199	718	1132	1175	4	1768	543	1659	96	556	2311
Postnatal care and home visit	1973	2.4	1079	0	830	583	1347	43	585	1388	1783	11	179	1973
Malaria (all types)	1949	2.3	388	523	984	1017	913	19	1520	429	1649	55	245	1949
Genito-urinary disorders and complaints	1820	2.2	2	81	1731	781	1035	3	1820		1715	47	59	1819
Gastro-intestinal disorders and complaints	1733	2.1	76	84	1564	645	1084	1	1733		1577	46	110	1730
Skin disorders	1240	1.5	302	329	603	691	547	1	1180	60	1054	35	151	1239
Malnutrition	729	0.9	705	22	0	351	372	6	59	670	256	70	403	729
Tuberculosis (all types)	561	0.7	6	36	516	285	269	6	488	73	442	26	93	560
Abortion	553	0.7	0	0	547	0	553	0	553		138	0	415	553
Musculoskeletal disorders	412	0.5	1	17	392	264	147	0	412		394	4	13	411
Eye problems	389	0.5	41	34	310	208	151	27	381	8	330	4	55	386
Hypertension	304	0.4	0	5	299	114	190	0	304		270	27	7	304
Obstetric and gynaecologic cases	270	0.3	0	3	265	0	269	0	270		233	1	38	269
Bacterial infection	366	0.4	267	30	63	179	185	2	154	212	236	30	100	366
Ear and mastoid disorders	185	0.2	87	23	73	83	99	2	152	33	149	5	31	184
Sexually transmitted infections or human immunodeficiency virus	185	0.2	0	5	175	72	108	0	185		177	5	2	180
Dental and oral disorders	83	0.1	10	5	68	43	40	0	82	1	75	3	5	83
Anaemia	51	0.1	3	1	47	12	39	0	51		47	1	3	51
Other	1613	1.9	138	193	1267	602	1002	8	1454	158	1432	45	135	1612

### Caseloads in children

Of the 16,331 cases of children below 5 years of age, 42.9% (7010 of 16,331) visited the health facilities for immunisation, and 35.9% (2520 of 7010) of them were fully immunised. The annual immunisation utilisation rate was 56 (95% CI: 55–58) visit per 100 children per year (7010 visits for an estimated average of 12,446 children in the actual cohort in a single year). For neonates, 6.7% (1088 of 16,331) came for assessment of danger signs and weight measurements. The rest, 50.4% (8233 of 16,331 cases), visited the health facilities for different types of illnesses and complaints. Out of these, more than 87% (7184 of 8233) were registered as new visitors. The most common registered diagnosis among children who visited the health facilities was pneumonia, contributing to 2635 of 7184 (36.7%) of the caseloads (Table 4).

**Table 4:** Prevalence of the ten most common disease diagnoses of under-five years of age children in Dale and Wonsho districts primary health care units, in 2018, Sidama, Ethiopia (*N* = 7184)

Diagnosis	Total cases Number (%)	Number of cases in Wonsho district	Number of cases in Dale district	Total under-five years children in both districts	Prevalence rate/1000 population
Pneumonia^a^	2635 (36.7)	996 (45.8)	1639 (32.7)	62,228	42.3
Diarrhoea^b^	1281 (17.8)	467 (21.5)	814 (16.3)	62,228	20.6
Other respiratory problems^c^	830 (11.6)	68 (3.1)	762 (15.2)	62,228	13.3
Febrile illnesses^d^	502 (7.0)	96 (4.4)	406 (8.1)	62,228	8.1
Malaria^e^	376 (5.2)	0 (0)	376 (7.5)	62,228	6.0
Malnutrition^f^	350 (4.9)	169(7.8)	181(3.6)	62,228	5.6
Skin problems^g^	257 (3.6)	96(4.4)	161 (3.2)	62,228	4.1
Parasitic and helminthic infections^h^	253 (3.5)	94 (4.3)	159 (3.2)	62,228	4.1
Bacterial infections^i^	273 (3.3)	88 (4.0)	147 (2.9)	62,228	4.4
Accidents^j^	78 (1.0)	47 (2.2)	31 (0.6)	62,228	1.3
Other	387 (5.4)	56(2.6)	331 (6.6)	62,228	6.2

^a^Pneumonia = all types of pneumonia; ^b^Diarrhoea = dehydration and all types of diarrhoea; ^c^Other respiratory problems = upper and lower respiratory disorder; ^d^Febrile illnesses = all acute febrile illnesses; ^e^Malaria = all types of malaria; ^f^Malnutrition = Severe acute and moderate malnutrition, and underweight; ^g^Skin problems = specific and unspecific skin problems; ^h^Parasitic and helminthic infections = all types of helminths, parasites; ^i^Bacterial infection = local and unspecific bacterial infection and sepsis; ^j^Accidents = all kinds of injury, trauma and road traffic accidents

### Service provision by sex

From the departments giving services to both genders, in adult OPD there were more female patients (54%) than men, whereas in emergency OPD, more than two-thirds (68.0%) of the patients were male. In the community health day services (CHD), in which services like vitamin A supplementation, de-worming and malnutrition screening are given to the community, the majority (82%) were female (Table 7 is attached as additional file 4).

### Health professionals’ opinions on health service utilisation

Table 5 shows the opinions of health officials in terms of improving health service utilisation. The health professionals’ opinions were categorised into seven thematic areas for local and regional health professionals separately. These themes were: budget allocations and alternative sources, sustainable drug and material supply, health workers capacity building, quality of services, access of health facilities, public and stakeholders involvement and collaboration, and supervision and record keeping.

**Table 5:** Solutions and recommendations forwarded by health professionals in Sidama region on the health service utilisation of Dale and Wonsho districts, 2018, Sidama, Ethiopia

**Recommendation from district health officials and health centre managers**	**Recommendation from Sidama Regional Health Bureau**
**Theme 1: Budget allocations and alternative sources**	
Ensuring and creating awareness on the community-based health insurance scheme. *“Most of the rural community thinks why would I have the community-based insurance, I might not be sick at all. But in reality our community spends more out of pocket.... Therefore, if we could convince and create awareness about the insurance, the frequency of the visit will increase.”*	
Allocating enough budgets for health facilities; and support those people who are very poor and orphans, and unable to pay for health services through healthcare financing. *“The kebeles should identify genuinely those who are the “poorest of the poor” and provide all services free of charge. Most people don’t come to health facility due to financial issue.”*	
**Theme 2: Sustainable drug and material supply**	
Supplying and ensuring availability of medical supplies or equipment like essential medications, laboratory reagents (diagnostic materials), physical examination apparatuses to provide quality health service. *“The main problem I think is lack of medications in the health centres, because of repeated encounter to get some of the medications from us, the community tends to go to other facilities.”*	Ensuring availability of different laboratory tests with high specificity; including hematologic tests for accurately identifying anaemia, diabetes, other infections and febrile illnesses; blood chemistry; urine analysis and culture. *“At the primary health level the availability and quality of laboratory tests in doubtful. The tests, if available, lack specificity. They mostly rely on making diagnosis based on signs or symptoms, and this could lead to misdiagnose or make wrong diagnosis”*
*“We don’t have functioning blood pressure measurement apparatus, such issues makes it to make accurate diagnosis.”*	Shortage of drugs and other supplies may affect service utilisation, because patients may prefer private clinics rather than government health facilities for these reasons. *“One of the issues here is if someone didn’t get drugs or laboratory test from the nearby health centre, he or she will go to other places.”*
**Theme 3: Health workers capacity building**	
Creating a compassionate, respectful and caring (CRC) health workforce by giving training and supervision to improve their attitudes. *“Training on CRC will improve the health professionals attitude and patients need such services”*	Augment the knowledge and skill of health workers through short- and long-term training.
Preparing manuals and give training on different components like community mobilisation. *“Health extension workers need more training so that they can equip the community about the services being given at health post and health centre level”* *“Strengthening integrated community case management, community-based newborn care, and integrated management of neonatal and childhood illness services are important to improve the diagnostic capacity.”*	To improve the diagnostic capacity of health facilities, further training, provision of more laboratory technicians and test kits may be recommended. *“Training whether short term or long term will improve the knowledge and skill of the health professionals, to make accurate diagnoses”*
Provide counselling and on-the-job and refreshment training to health professionals to halt negligence or malpractice by health professionals. *“Regular counselling and refreshment trainings should be given to health professionals, this will help in improving malpractices in their day to day practice”*	
**Theme 4: Quality of services**	
	Working to make the health facilities function per the standards will increase the quality of care, which intern will improve the utilisation. *“For example, stabilisation centre service utilisation for severely malnourished children is low. This may be due to the fact that not all health centres and hospitals are providing services as per standard due to shortage of meals for caretakers, lack of trained staff and shortage of stabilisation centre kits. Therefore, working to mitigate these factors in collaboration with other stakeholders might be a solution.”*
**Theme 5: Access of health facilities**	
Strengthening linkages between the district health offices and health centres, and between health centres and health posts, by creating timely and smooth lines of communication.	Building maternity waiting rooms will improve access related problem. *“The lower postnatal care utilisation shows that almost all primary health care units are not keeping mothers at the health facilities at least for 24 h after delivery. This may be due to a shortage of maternity waiting rooms because most government health facilities have incomplete premises and infrastructure.”*
Plan to increase the number of health facilities. *“Due to the geographical inaccessibility of our woreda (Wonsho), more health facilities should be constructed to increase utilisation”*	Some health facilities are not accessible throughout the week for 24 h. *“Even though health-seeking behaviour of the community was improved, most health posts may be closed during working hours and health centres do not provide some services the entire week. Therefore, ensuring delivery of service at all times could increase utilisation.”*
**Theme 6: Public and stakeholders involvement and collaboration**	
The public should be involved from planning to implementation of health services. The health extension workers also should focus on creating awareness on health care seeking behaviour. Every gathering should be taken as a venue for awareness creation. Moreover, collaboration with other non-governmental organisations might alleviate problems with capacity building, material supply and infrastructure. *“Involving women health development armies, who are volunteer community health workers responsible for mobilising the community, during planning and training them on implementation issues.”* *“Encourage health extension workers to strictly work on house-to-house visits and educating the community about health service utilisation.”* *“Giving health education about health services in different locations like health facilities, schools, community gatherings* etc.*”* *“Creating and strengthening communication with other stakeholders like non-governmental organisations.”*	
**Theme 7: Supervision and record keeping**	
Enhance recording and reporting system by giving on-the-job and refreshment training and supportive supervision.	
Timely supportive supervision, follow-up and feedback (monitoring and evaluation) should be strengthened to enhance the commitment of health professionals. *“We have missed information due to poor documentation and negligence; anything not recorded is considered as not being done. Therefore, training and supervisions should focus on these areas too.”*	

## Discussion

This study shows a low health service utilisation rate in one of the most densely populated areas of Ethiopia (Sidama), especially among the rural population. Febrile illnesses were prevalent diseases, and family planning cases constituted the largest proportion of the recorded diagnoses. Yet, very few non-communicable diseases were diagnosed.

Health professionals accountable for the delivery of health services in the area suggested improving the low health service utilisation by improving the quality, affordability, and accessibility of health services, improving the health professionals’ skills, and introducing better diagnostic modalities. However, further studies are desired to find out if the involvement of health workers may possibly result in improved resource allocations and enhanced services.

A key strength of this study is the inclusion of all primary health care units covering the urban and rural populations. The data for the services were collected for a period of a whole year, for all types of services given, and included all governmental health centres and health posts. In addition, the comments and suggestions of health officials to improve health service utilisation were included as part of the study.

This study is not without limitations. Due to the loss of some data in three of the health centres, there might be some information bias, and there could be some under-reporting for delivery and eye services. However, given the large study population, the proportion of missing data from these units is small, and this information bias is therefore probably negligible. There were also missing data on visit information. However, the number of missing cases was few compared to the total cases, therefore, they would not change the results. In addition, children who started their vaccination in previous fiscal year were recorded as repeat. This might cause under-reporting in vaccination coverage.

Even though the involvement of the health professionals in the research process was a new and a good experience, the political context might have introduced methodological biases. A social-desirability bias in which the health professionals responded by considering the expectations of the researchers is one of the potential biases in a country where a bureaucratic and hierarchical structure prevails. The health professionals might not have critically evaluated the day-to-day services at their institution by considering the results from the study. Instead, their focus was mainly on general, administrative, and structural solutions, overlooking some of the findings that required local or institutional recognition [40].

Moreover, the diagnosis at primary health care units was mainly based on clinical findings, as very few diagnoses were supported by laboratory findings at the health centres. Thus, the lack of laboratory support and clinical skills to diagnose, for example, typhoid fever, typhus, anaemia, diabetes mellitus, malignant disorders, and hypertension, may explain why such diseases apparently occurred rarely. There were also a few cases that came to seek health services from other areas outside the study districts, and their population was not considered in the denominator during the calculation. We assume that some people from our study districts might seek care outside their catchment. People who primarily went to private clinics and hospitals farther away were, however, not recorded, and this may be a cause of under-reporting of use of health services in the area.The overall health service utilisation rate expressed in our study is higher than the rate of primary healthcare visits in central, southern and western Ethiopia recorded 30 years ago [41]. Yet, in our study, the utilisation rate is far lower than the targeted individual outpatient visits per year of the country and of other community-based studies in Oromia and Amhara regions [5, 20, 42]. This may partly be due to the methodological difference between the studies, closure of health posts for some periods during working hours and inability to provide some services by the health centres for the entire week [43]. Patients may also prefer traditional medicine or self-treatment [42], private clinics or self-referral to higher-level health facilities outside their catchment area. This might be due to poor quality health services in government health facilities, such as the unavailability of drugs and laboratory tests, as well as unskilled staff or an unwelcoming attitude of health professionals [13, 43, 44].

Our study indicated that the urban population used the health services more than their rural counterparts, as has been reported by others [42, 45]. This urban-rural difference might be attributed to a higher poverty, lower accessibility and lower education for the rural population [46].

The health service utilisation by children and older age people is lower than for adult people in our study, as has been reported by others [42]. One of the explanations for this may be because these age groups are usually dependent on other people to transport them to the health facilities. Another explanation could be that older people still rely more on traditional medicine [47]. Furthermore, the unavailability of some child health services for several days of the week could also have reduced the number of cases in these groups [43].

Our study showed that more people visited health centres than health posts. This finding is in agreement with other studies done in Ethiopia and Zambia showing preference of health centres over hospitals, health posts and private facilities [21, 48]. This might be due to the proximity of health centres to the community, besides the limited services availability and absence of qualified professionals at the health posts [13]. Moreover, the community often considers the health extension workers to be involved mainly in preventive health services, such as health education and sanitation, than the curative services [49].

Consistent with our finding, some studies showed a higher health service utilisation by women than men [20, 50]. The possible explanation for this might be the reproductive needs of women requiring separate services than men. Since most women take their children to health facilities, they might also get services or treatment for themselves [20]. The other reason might be the higher self-medication tendency of men than women [21].

The most common causes of morbidity in this study were consistent with the national study of health and health-related indicators in 2015 [51]. Diarrhoeal diseases were at the top of the list on a study done 30 years ago [41]. However, among children below 5 years of age, pneumonia was the major cause of morbidity in our study [51]. The recorded causes of morbidity may also be inaccurate due to the inadequate knowledge or skills of health professionals which may have contributed to incorrect diagnoses or over-diagnosis [52].

Family planning is the main maternal and child health service registered in our study. This finding is lower than for other sub-Saharan countries but similar to other studies done in Ethiopia [53–55]. The lower fertility rate found in the area documented in a recent study might also be an indication of improved family planning usage [56]. Besides, family planning services are available free of charge in governmental health facilities.

Antenatal care utilisation in our study was lower than most local and national studies done in the country except for pastoral regions [57–59]. Similarly, delivery service utilisation in this study is higher than other studies done in the country but lower than the national study of health facility delivery [58–60]. This difference might arise because our study used health facility data, whereas the other studies were community based.

Postnatal care was much lower than for other maternal health services but is similar to other national and regional studies [57, 58, 61]. This might be due to the absence of maternity waiting rooms in almost all primary health care units to admit the mother for at least 24 h.

Even though recent studies showed an increase in the prevalence of hypertension, there were few cases of hypertension in our study [62–64]. The discrepancy in the results might be due to the inability or lack of skills or materials to make an accurate diagnosis at the primary health care units. Similarly, in the last national Ethiopian demographic and health survey (EDHS) report, the prevalence of anaemia in women of reproductive age has increased to 24% and to 57% in children [65]. However, in our study, there were very few cases with anaemia. This might be attributed to the inability to measure haemoglobin at the institutions in the study area. Likewise, the number of diabetes mellitus cases diagnosed in our study area was negligible. Similar to hypertension and anaemia, the prevalence of diabetes is most likely under-diagnosed due to the lack of clinical skills and diagnostic materials at the institutions.

## Conclusion

The Ethiopian government aims to enhance the coverage of primary healthcare services in the rural communities [5]. However, there is low and unequal health service utilisation in Ethiopia. We observed low annual health service utilisation rate among rural residents, men, children and elderly, and health post users. Among the reported cases, family planning constituted the largest proportion, and non-communicable diseases were the least diagnosed group. Pneumonia was the most common recorded diagnosis among children younger than 5 years of age. Even if there is no complete agreement between registry data and the health professionals’ opinions, most of the ideas overlap and give direction to improving health service utilisation. The findings of this study will help in the effort the government is undertaking to make quality health services accessible and affordable to the underserved population. Ensuring availability of drugs or medical supplies, laboratory tests and standard working procedure could increase the service usage. Moreover, supportive supervision needs to be provided, and the knowledge and skills of healthcare professionals and their diagnostic capacity need further improvement. In addition, the quality of health services should also be assessed and given priority so that the health services can be utilised by every segment of the population.

## Supplementary Information

**Additional file 1.** List of registers.

**Additional file 2.** Operational definitions.

**Additional file 3.** Treatment at departments.

**Additional file 4.** Service provision by sex.

## Data Availability

The datasets used and/or analysed during the current study are available from the corresponding author on reasonable request.

## Abbreviation

OPD: Outpatient department
