# Supplementary material for: Low and unequal use of outpatient health services in public primary health care facilities in southern Ethiopia: a facility-based cross-sectional study
Source: BMC Health Serv Res. 2021 Aug 6;21:776. doi: 10.1186/s12913-021-06846-x (PMC8344135; doi:10.1186/s12913-021-06846-x)
Supplement: Supplementary file 1 — Additional file 1. List of registers. [file 12913_2021_6846_MOESM1_ESM.doc]

**Additional file 1: List of registers**

Table 8: List of registers reviewed to extract the data from health centres and health posts in Dale and Wonsho districts, 2017/18, Sidama, Ethiopia

| **Registers from health centres** | **Registers from health posts** |
| --- | --- |
| ANC register | ANC register |
| Delivery register | Family planning register |
| Family planning register | PNC register |
| Infant immunization and growth monitoring register | TB suspected case screening and treatment register |
| PNC register | Malaria Assessment |
| Cervical cancer screening and prevention register | Infant immunization |
| Safe/Post abortion Care register | ICCM 0-2 months |
| Comprehensive abortion care register | ICCM 2 months-5 years |
| Outpatient department (OPD) register/ OPD abstract register | Registration book for therapeutic feeding |
| Emergency department register | Community health day services (CHD) register |
| Unit TB register | Forms below are from family folders   - Integrated Antenatal, Labor, Delivery, Newborn and Postnatal Card - Health card: FP and Immunization |
| Integrated under-five registration book : from birth to 2 months |
| Integrated under-five registration book : from 2 months up to 5 years |
| Stabilization centre and Outpatient therapeutic feeding program register |

Table 8: List of registers reviewed to extract the data from health centres and health posts in Dale and Wonsho districts, 2017/18, Sidama, Ethiopia (continued)

| **Registers from health centres** | **Registers from health posts** |
| --- | --- |
| Referral register | - Health posts disease information tally |
| Diabetes and Hypertension treatment register |  |
| Eye clinic registration |  |
| - Some health facilities registered TT and PMTCT separately from ANC registers |  |

NB: The information extracted from each registers were, name of the district, name of health facility, address of the health facility (rural or urban), unit or department, card number, date of visit, sex, age (in days, months, years or date of birth), address (kebele), diagnosis, and visit type.
