# Supplementary material for: Low and unequal use of outpatient health services in public primary health care facilities in southern Ethiopia: a facility-based cross-sectional study
Source: BMC Health Serv Res. 2021 Aug 6;21:776. doi: 10.1186/s12913-021-06846-x (PMC8344135; doi:10.1186/s12913-021-06846-x)
Supplement: Supplementary file 2 — Additional file 2. Operational definitions. [file 12913_2021_6846_MOESM2_ESM.doc]

**Additional file 2: Operational definitions**

Health service utilisation: Defined as the use of the existing modern health services per year in primary health care units by individuals for their psychological, physical and social health problems, whether this is for preventive or curative purpose, measured as the quantity of health care services used (1–3)

Antenatal care: Care given to pregnant women by a skilled provider expressed by the number of visits to the health facility during her last pregnancy (4).

Immunisation: A service given to a child for all recommended vaccines, including Bacillus-Calmette-Gueri (BCG), Diphtheria-Pertussis-Tetanus, Hepatitis B and Haemophilus influenza type B (Penta-valent vaccine), Pneumococcal Conjugated Vaccine (PCV), Oral Polio Vaccine (OPV), Monovalent human rotavirus vaccine (RV1), Inactivated Polio Virus (IPV) and Measles vaccines. In addition, Tetanus Toxoid (TT) vaccine for pregnant women (4,5).

Skilled delivery: Births delivered with the assistance of doctors, nurse or midwives, health officers, and health extension workers (4).

Postnatal care: Care given to the mother and the newborn within 24 hours, day 3 (48-72 hours), between days 7 and 14, and six weeks after birth with integration of home visits (6).

Outpatient utilisation rate: The number of new outpatient visits to health facilities per year relative to the total population of the same geographical area (2).

We calculated the utilisation rates for each variable as: The number of disease events divided by the eligible population of the same geographic area. We estimated the proportion of eligible population by multiplying the total population of the districts by the percentage of a particular category obtained from census and surveys.

Departments/Units: Structures of health facilities used to deliver standard health services in an organised manner (7).

New visitors were defined as those patients who attended the health facility for the first time (8). Repeat visitors were those who attended the health facility for multiple times for the same diagnoses/illness within the reporting period, one Ethiopian fiscal year (July to June), recorded once as new visit previously (8).

For family planning service: “New acceptors” refers to the number of modern contraceptive method acceptors who receive family planning services from a recognised family planning providing facility for the first time irrespective of the method used. Each such acceptor was counted once. Each “repeat acceptor” is counted once, irrespective of number of times family planning services were received during that fiscal year (8).

For immunisation services, new visitors were children who received their first dose of vaccines and repeat visitors were those who received more than one dose in one year.

**References**

1. Ethiopian Federal Ministry of Health. Health Sector Transformation Plan (2015/16-2019/20). Addis Ababa, Ethiopia: Federal Ministry of Health; 2015.

2. World Health Organization. Monitoring the Building Blocks of Health Systems: a Handbook of Indicators and Their Measurement Strategies. Vol. 35. Geneva, Switzerland:World Health Organization; 2010.

3. Dagnew T, Tessema F, Hiko D. Health service utilization and reported satisfaction among adolescents in Dejen District, Ethiopia: a cross-sectional study. Ethiop J Health Sci. 2015;25(1):17–28.

4. Ethiopian Public Health Institute (EPHI) [Ethiopia] and ICF. Mini Demographic and Health Survey 2019: Key Indicators. Rockville, Maryland, USA; 2019.

5. Federal Ministry of Health. National Expanded Programme on Immunization Comprehensive Multi-Year Plan 2016 - 2020. Addis Ababa: FMoH; 2015.

6. World Health Organization. Postnatal Care for Mothers and Newborns Highlights from the World Health Organization 2013 Guidelines. Geneva, Switzerland:World Health Organization; 2015.

7. Ethiopian Standards Agency (ESA). Ethiopian standard Health Center- Requirements. First edit. Addis Ababa, Ethiopia: Ethiopian Standards Agency; 2012. 1–126 p.

8. Federal Ministry of Health. HMIS INDICATOR REFERENCE GUIDE Technical Standards : Area 1. Addis Ababa, Ethiopia; 2017.
