# Supplementary material for: Low and unequal use of outpatient health services in public primary health care facilities in southern Ethiopia: a facility-based cross-sectional study
Source: BMC Health Serv Res. 2021 Aug 6;21:776. doi: 10.1186/s12913-021-06846-x (PMC8344135; doi:10.1186/s12913-021-06846-x)
Supplement: Supplementary file 3 — Additional file 3. Treatment at departments. [file 12913_2021_6846_MOESM3_ESM.doc]

**Additional file 3**: Treatment at departments

|  | **Unit/Department** | **Number of cases** | **%** |
| --- | --- | --- | --- |
| 1. | Adult OPD | 24,388 | 30.1 |
| 2. | Family planning (FP) | 22,423 | 27.6 |
| 3. | Expanded Program of Immunisation (EPI) | 7,010 | 8.6 |
| 4. | Under 5 years OPD | 6,316 | 7.8 |
| 5. | Delivery | 5,659 | 7.0 |
| 6. | Antenatal care (ANC) | 4,482 | 5.5 |
| 7. | Cervical cancer screening | 2,289 | 2.8 |
| 8. | Antenatal care with Tetanus Toxoid immunisation (ANC with TT) | 1,828 | 2.3 |
| 9. | Emergency OPD | 1,578 | 2.0 |
| 10. | Postnatal care | 1,442 | 1.8 |
| 11. | Under 2 months OPD | 984 | 1.2 |
| 12. | TB clinic | 565 | 0.7 |
| 13. | Tetanus Toxoid (TT) | 492 | 0.6 |
| 14. | Abortion | 521 | 0.6 |
| 15. | Outpatient Therapeutic Feeding Program (OTP) | 496 | 0.6 |
| 16. | Prevention of mother-to-child transmission (PMTCT) | 341 | 0.4 |
| 17. | Eye clinic | 156 | 0.2 |
| 18. | Community health day services (CHD) | 126 | 0.2 |
| 19. | Stabilisation centre (SC) | 33 | 0.04 |
|  | Total | 81,129 | 100.0 |

Table 6: Distribution of cases per unit or department in Dale and Wonsho district primary health care units in 2018, Sidama, Ethiopia (N=81,129)
