# Supplementary material for: Low and unequal use of outpatient health services in public primary health care facilities in southern Ethiopia: a facility-based cross-sectional study
Source: BMC Health Serv Res. 2021 Aug 6;21:776. doi: 10.1186/s12913-021-06846-x (PMC8344135; doi:10.1186/s12913-021-06846-x)
Supplement: Supplementary file 4 — Additional file 4. Service provision by sex. [file 12913_2021_6846_MOESM4_ESM.doc]

**Additional file 4**: Service provision by sex

| Unit /Department | Sex | |  | Total |
| --- | --- | --- | --- | --- |
|  | Female | Male | Missing |  |
|  | Number (%) | Number (%) | Number (%) | Number (%) |
| Adult OPD | 13,129 (53.8) | 11,220 (46.0) | 39 (0.2) | 24,388 (100.0) |
| Emergency OPD | 475 (30.1) | 1,078 (68.3) | 25 (1.6) | 1,578 (100.0) |
| Under 5 years OPD | 2,976 (47.1) | 3,304 (52.3) | 36 (0.6) | 6,316 (100.0) |
| Under 2 months OPD | 468 (47.6) | 514 (52.2) | 2 (0.2) | 984 (100.0) |
| TB clinic | 271 (48.0) | 288 (51.0) | 6 (1.1) | 565 (100.0) |
| EPI | 2,932 (41.8) | 3,188 (45.5) | 890 (12.7) | 7,010 (100.0) |
| OTP | 250 (50.4) | 245 (49.4) | 1 (0.2) | 496 (100.0) |
| SC | 21 (63.6) | 8 (24.2) | 4 (12.1) | 33 (100.0) |
| Eye clinic | 56 (35.9) | 74 (47.4) | 26 (16.7) | 156 (100.0) |
| Community health day services | 103 (81.8) | 20 (15.8) | 3 (2.4) | 126 (100.0) |

Table 7: Distribution of cases by sex in departments serving both genders in Dale and Wonsho districts, 2018, Sidama, Ethiopia (N=41,156)
